# Endosomal recycling inhibitors downregulate estrogen receptor-alpha and synergise with endocrine therapies

**DOI:** 10.1007/s10549-023-07225-2

**Published:** 2024-01-16

**Authors:** Kelsey A. Fletcher, Mai H. Alkurashi, Andrew J. Lindsay

**Affiliations:** Membrane Trafficking and Disease Laboratory, School of Biochemistry & Cell Biology, Biosciences Institute, University College Cork, Cork, T12 YT20 Ireland

**Keywords:** Estrogen receptor, Androgen receptor, Receptor recycling, Small molecules, HER3

## Abstract

**Purpose:**

Breast cancer (BC) accounts for roughly 30% of new cancers diagnosed in women each year; thus, this cancer type represents a substantial burden for people and health care systems. Despite the existence of effective therapies to treat BC, drug resistance remains a problem and is a major cause of treatment failure. Therefore, new drugs and treatment regimens are urgently required to overcome resistance. Recent research indicates that inhibition of the endosomal recycling pathway, an intracellular membrane trafficking pathway that returns endocytosed proteins back to the plasma membrane, may be a promising strategy to downregulate clinically relevant cell surface proteins such as HER2 and HER3, and to overcome drug resistance.

**Methods:**

To investigate the molecular mechanism of action of an endosomal recycling inhibitor (ERI) called primaquine, we performed a reverse-phase protein array (RPPA) assay using a HER2-positive breast cancer cell line. The RPPA findings were confirmed by Western blot and RT-qPCR in several BC cell lines. Novel drug combinations were tested by MTT cell viability and clonogenic assays.

**Results:**

Among the signalling molecules downregulated by ERIs were estrogen receptor-alpha (ER-α) and androgen receptor. We confirmed this finding in other breast cancer cell lines and show that downregulation occurs at the transcriptional level. We also found that ERIs synergise with tamoxifen, a standard-of-care therapy for breast cancer.

**Discussion:**

Our data suggest that combining ERIs with hormone receptor antagonists may enhance their efficacy and reduce the emergence of drug resistance.

**Supplementary Information:**

The online version contains supplementary material available at 10.1007/s10549-023-07225-2.

## Introduction

Breast cancer (BC) is the most common cancer type in women, and with an ageing population, its incidence is increasing [1]. Although rare, BC also occurs in men, accounting for approximately 1% of all cancers in men [2]. BC can be categorised according to the presence or absence of several receptors, allowing it to be classified into a number of subtypes. These include the estrogen receptor (ER), human epidermal growth factor receptor 2 (HER2), and progesterone receptor (PR). Luminal A and luminal B breast cancers both express ER. Luminal B-like breast cancer is HER2 positive and ER positive. Basal-like or triple-negative breast cancer (TNBC) does not express any of these receptors. The subtype influences diagnosis, prognosis, and treatment of the tumour [3].

ER is a nuclear hormone receptor that binds to estrogen in the cytoplasm and subsequently translocates into the nucleus to activate expression of estrogen responsive genes. 70–80% of BCs are ER-positive; thus, estrogen and ER are commonly targeted during treatment of this type of cancer. There are two isoforms of ER, alpha and beta, and activation of ER-α by estrogens is primarily responsible for enhanced proliferation in ER-positive BC. In contrast, ER-β has an antiproliferative effect and can counteract the tumorigenic effects of ER-α [4]. Tamoxifen is a selective estrogen receptor modulator (SERM) that binds ER and induces conformational changes in the receptor that inhibits coactivator binding, and thus, prevents its activation. While tamoxifen reduces the mortality rate in ER-positive BC, acquired resistance due to long-term use is estimated to occur in approximately one third of patients [5]. Hence, the risk of recurrence is of considerable clinical concern and leads to a need for novel and innovative ER-targeting treatments [6].

Androgen receptor (AR) is also a nuclear receptor and mediates the effect of androgens by modulating the expression of genes involved in proliferation and survival. Androgen hormones and their receptor play a vital role in normal prostate development, but androgen receptor (AR) is also the main driver in the development of prostate cancer [7].

AR also plays an important role in normal female biology including fertility and breast development. Moreover, it is expressed in 60–80% of breast tumours [8]. Nearly 90% of ER-positive tumours express AR, but less than 30% of ER-negative breast cancers are AR positive. Outcomes for patients with ER-positive/AR-positive tumours are better than those with ER-negative/AR-positive tumours. This is believed to be due to AR competing with ER for binding to estrogen response elements, resulting in impaired transcription of estrogen-regulated genes [9]. In contrast, in ER-negative breast cancers, AR signalling can drive tumour growth. ER activation has also been implicated in prostate cancer progression [10]. Thus, therapeutic strategies that modulate the levels of these nuclear receptors could prove effective at delaying or preventing breast cancer progression.

Recent work from our laboratory has discovered that inhibition of the endosomal recycling of the HER2 receptor tyrosine kinase (RTK) leads to downregulation of its signalling, and ultimately to its degradation in lysosomes [11]. Proteins in the plasma membrane are continuously internalised into organelles called early endosomes by a process called endocytosis. This is a means by which the cell controls the strength and duration of their signalling. From early endosomes, cell surface proteins can be returned to the plasma membrane along the endosomal recycling pathway to be re-used, or they are sent to lysosomes for degradation. The majority of endocytosed proteins are recycled back to the plasma membrane [12]. We reported that a small molecule inhibitor of endosomal recycling, called primaquine (PQ), reduces the total protein levels of HER2 and synergises with HER2-targeting therapies. Furthermore, we showed that BC cells with acquired or innate resistance to HER2-targeted therapies have enhanced sensitivity to PQ [11]. We demonstrated that when endosomal recycling is inhibited, internalised HER2 and its heterodimerisation partner, HER3, are diverted to lysosomes for degradation.

To gain a greater understanding of the mechanism of action of PQ, we performed a reverse-phase protein array (RPPA) assay to determine the effect that the drug has on the levels and activation status of approximately 450 proteins that have been implicated in cancer. Interestingly, we found that both ER-α and AR were downregulated in a HER2-amplified cell line that had been treated with PQ. Between 60 and 70% of HER2-positive breast cancers co-express hormone receptors and bidirectional crosstalk between the ER and HER2 signalling pathways has been well established in breast cancer [13]. This has clinical consequences as co-expression of these receptors modulates tumour response to both HER2-targeting and endocrine therapies [13]. Furthermore, activation of the ER signalling pathway has been reported as an escape mechanism for tumours that are subject to HER2 inhibition [14].

We confirmed these findings in other hormone receptor-positive BC cell lines. We also show that endosomal recycling inhibitors synergise with tamoxifen, a standard-of-care therapy for BC. These results suggest that modulating the activity of the endosomal recycling pathway may be a useful strategy for downregulating the activity of hormone receptors in BC.

## Materials and methods

### Reagents

All cell culture media and supplements were purchased from Sigma-Aldrich (UK). Monensin, resazurin, and MG132 were from Sigma-Aldrich, and plasticware was from Sarstedt. Primaquine, lapatinib, enzalutamide, and MTT were purchased from Carbosynth, UK. BafA1 was obtained from Merck.

Antibodies specific for ER-α (#8644), AR (#5153), and HER3 (#12708) were from Cell Signalling Technology. Mouse monoclonal anti-alpha tubulin (T5168) was from Sigma-Aldrich. 680RD-conjugated goat anti-Mouse (C80619-5) and 800CW-conjugated goat anti-Rabbit (C80426-08) secondary antibodies were from LI-COR Biosciences.

### Reverse phase protein array (RPPA)

BT474 cells were seeded in a 6-well plate at 5 × 10^5^ cells per well and cultured until the cells had reached 70–80% confluency. The medium was replaced with 2 ml fresh culture medium with or without 10 µM PQ. 24 h later the cells were washed twice with cold PBS and lysed in lysis buffer (1% TX-100; 50 mM Hepes, pH 7.4; 150 mM NaCl; 1.5 mM MgCl_2_; 1 mM EGTA, 100 mM NaF; 10 mM Na pyrophosphate, 1 mM Na_3_VO_4_; 10% glycerol) plus protease inhibitors. Cell and nuclear debris were removed by centrifugation at 20,000 x*g*. The protein concentration was determined by Bradford assay and adjusted to 1.5 µg/µl with lysis buffer and mixed with 4X Sample buffer plus β-mercaptoethanol, but without bromophenol blue. The cells were heat denatured, snap-frozen, and two biological replicates for each condition were sent on dry ice to the MD Anderson Cancer Center RPPA Core Facility (TX, USA) for RPPA analysis. Protein levels were determined by interpolation of dilution curves to give log2 values. The data were then normalised for protein loading and transformed to linear values. Normalised linear values were transformed to log2 values, and heat maps were generated using Java Treeview.

### Cell culture

The human breast cancer cell line MCF-7 (ATCC) was cultured in Dulbecco’s Modified Eagle Medium (DMEM). MDA-MB-134 VI and SUM44-PE cells were cultured in RPMI 1640 media. Authenticated BT474 cells were purchased from CalTag Medsystems and cultured in DMEM. Media were supplemented with 10% foetal bovine serum, 1% L-glutamine, and 1% penicillin–streptomycin. Cells were cultured at 37 °C in a humidified atmosphere at 5% CO_2_. All cell lines were regularly checked for mycoplasma contamination by fluorescence microscopy using DAPI staining or with MycoAlert Mycoplasma Detection Kit (Lonza).

To knockdown HER3 expression, two independent siRNA duplexes were purchased (Sigma). A siRNA duplex targeting firefly luciferase was used as a negative control (siFLUC). Reverse transfections were performed with a final siRNA concentration of 20 nM using Lipofectamine RNAiMax (Thermo Fisher), according to the manufacturer’s instructions. Cell lysates were prepared 72 h post-transfection.

### Cell viability assays

#### MTT assay

The MTT assay was used to assess cell viability of cells grown in 2D. 72 h post-drug treatment, MTT (Carbosynth, UK) was added to the cells and incubated for 2–3 h at 37 °C. The cells were solublised with MTT solvent (4 mM HCl, 0.1% NP-40 in isopropanol). The OD_570_ and OD_630_ was measured on a MultiSkan Go plate reader (Thermo Scientific). Quadruplicate wells for each treatment were analysed, and each experiment was carried out at least 3 times.

#### IC50 and synergy

The IC_50_ values were determined by nonlinear regression of the dose–response data using GraphPad Prism 9.4.1 for PC (GraphPad Software, La Jolla, CA). Synergy was determined by the method of Chou and Talalay [15, 16]. In brief, cells were exposed to 1:1 ratios of the IC_50_ values of hormone antagonist and endosomal recycling inhibitor at 1/8 × IC_50_, ¼ × IC_50_, ½ × IC_50_, IC_50_, 2 × IC_50_, and 4 × IC_50_. Cell viability was determined after 72 h treatment, and the CI was calculated using the CompuSyn software [17], to determine the presence of synergism (CI < 1) or antagonism (CI > 1).

#### Clonogenic assays

10,000 MCF-7 cells were seeded into each well of a 12-well plate. After 24 h, medium was replaced with fresh medium containing the respective drug. Media/drug solution was replaced every 3–4 days. Plates were incubated for a total of 10 days. Following drug treatment, the cells were washed with warmed PBS and living cells were stained with 0.5% crystal violet in 20% methanol. Plates were scanned on a flatbed scanner, and ImageJ was used to quantify colonies.

### Immunoblot analysis

Whole cell lysates were prepared in RIPA buffer supplemented with phosphatase and protease inhibitors (10 mM NaF + 1 mM NaOV + 1X PIC + 10 mM Na.pyrophosphate + AEBSF + 10 mM β-glycerophosphate). Cells were incubated in the RIPA solution on ice for > 20 min. Lysates were centrifuged at 14,000 rpm to remove cellular and nuclear debris. Bradford assay was used to determine protein concentration, and samples were heat denatured for 5 min at 95 °C in 4X loading buffer.

SDS-PAGE was used to resolve equal amounts of proteins, and the proteins were transferred to nitrocellulose. Revert 700 Total Protein stain (LI-COR Biosciences, UK) was used to reversibly stain the nitrocellulose membranes. Membranes were scanned using the Odyssey Infrared Imaging System (LI-COR Biosciences, UK). After washing off the Revert, membranes were blocked with Odyssey Blocking Buffer TBS at room temperature for approximately one hour. Primary antibodies were diluted in Odyssey Blocking Buffer TBS, and membranes were incubated with the primary antibody at 4 °C overnight. IRDye-conjugated secondary antibodies were used for detection with the Odyssey system. Secondary antibody incubation was carried out for one hour. LI-COR Image Studio software was used for densitometry, and bands were normalised against the Revert 700 stain for that sample, according to the manufacturer’s instructions.

### RT-qPCR

100,000 MCF-7 cells/well were seeded in a 12-well plate. After 48 h, wells were treated for 24 h with the appropriate drug in 1 ml media. Total RNA was extracted using the GenElute Mammalian Total RNA Miniprep Kit (Sigma-Aldrich, #SLCD5747). The extracted RNA samples were reversed transcribed into cDNA using the QuantiTect Reverse Transcription Kit (Qiagen, #163,017,287). The cDNA obtained was then quantitatively amplified using real-time PCR with FastStart Essential DNA Green Master (Roche) ready-to-use SYBR Green I reaction mix on a Roche LightCycler 96 instrument.

### Cancer genomics

*ERBB2* (HER2), *ERBB3* (HER3), *ESR1* (ER-α), and *AR* gene expression in the 68 breast cancer cell lines available in the Cancer Cell Line Encyclopedia (CCLE) were determined, and the median gene expression for *ERBB3* or *ERBB2* was used to divide the datasets into high (above median) and low (below median) expressing groups. The same strategy was used to analyse 1903 breast tumours from the METABRIC study available in the cBioPortal database [18, 19].

The protein levels of ER-α, AR, HER2, and HER3 in breast cancer cell lines available in the DepMap Portal database were downloaded and imported into GraphPad Prism where they were subjected to a simple linear regression analysis.

### Data analysis

Statistical significance was determined using the Student’s *t*-test, or where specified a 1-way analysis of variance, using GraphPad Prism. Significance was classified as a *P*-value of * < 0.05, ** < 0.01, *** < 0.001.

## Results

### Primaquine downregulates the estrogen and androgen receptors

**Fig. 1:**
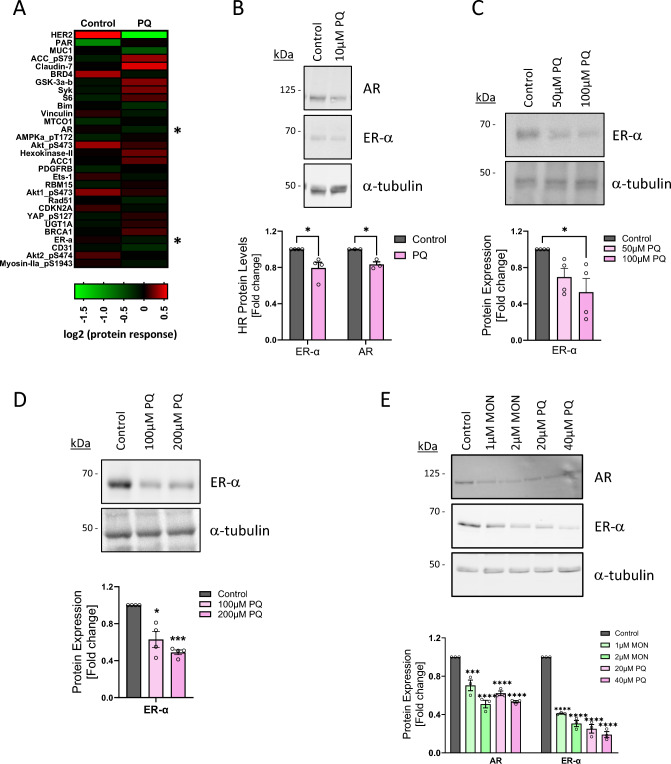
Endosomal recycling inhibitors downregulate ER-α and AR. **A** Heatmap displaying the top 30 hits from the RPPA analysis of control and 10 µM PQ-treated BT474 cells (mean of two biological replicates; * indicates AR and ER-α). **B** Lysates of BT474 cells treated with 10 µM PQ for 24 h were immunoblotted with the indicated antibodies. Quantitative analysis of the fold difference of hormone receptor protein levels relative to control untreated cells indicated in the histogram (*n* = 3–4). MDA-MB-134 VI (**C**) and SUM44-PE (**D**) invasive lobular breast carcinoma cells were treated with the indicated concentrations of PQ for 24 h prior to lysis and immunoblotting. Histograms indicate the fold change in ER-α protein levels relative to untreated cells (*n* = 4). **E** MCF-7 cells were treated with the indicated concentrations of PQ and MON for 24 h. Lysates were immunoblotted with ER-α and AR antibodies, and the histogram indicates the fold difference in protein levels relative to untreated cells (*n* = 3)

The BT474 HER2-amplified breast cancer cell line was used to systematically determine the effect of the endosomal recycling inhibitor PQ on cellular signalling networks. Control and PQ-treated BT474 cell lysates were analysed by reverse-phase protein array (RPPA). The top hit was HER2, thus, validating the assay (Fig. 1A). As expected, several proteins and pathways that potentiate HER2 oncogenic signalling were downregulated, including components of the PI3K/Akt/mTor signalling pathway. Interestingly, the hormone receptors ER-α and AR were also downregulated (Fig. 1A). To confirm these findings, lysates from BT474 cells treated with or without 10 µM PQ were analysed by Western blot. An approximately 20% reduction in ER-α protein levels was consistently observed (Fig. 1B). PQ treatment also led to a reduction in the levels of ER-α in the MDA-MB-134 VI and SUM44-PE BC invasive lobular breast carcinoma cell lines (Fig. 1C, D). Both of these cell lines express ER-α but have negligible amounts of AR (Fig. S2A).

We then switched to the canonical hormone receptor-positive MCF-7 cell line which expresses both ER-α and AR (Fig. S2A). MCF-7 cells were treated with IC_50_ and IC_75_ concentrations of PQ and a second ERI called monensin [20]. PQ treatment resulted in a 75–80% reduction in the total protein levels of ER-α, and monensin resulted in a 60–70% reduction (Fig. 1E). PQ and monensin induced similar, but more modest, reductions in AR (Fig. 1E). Thus, endosomal recycling inhibitors can downregulate ER-α and AR in multiple HR-positive BC cell lines.

### Endosomal recycling inhibitors downregulate ER-α at the transcriptional level

**Fig. 2:**
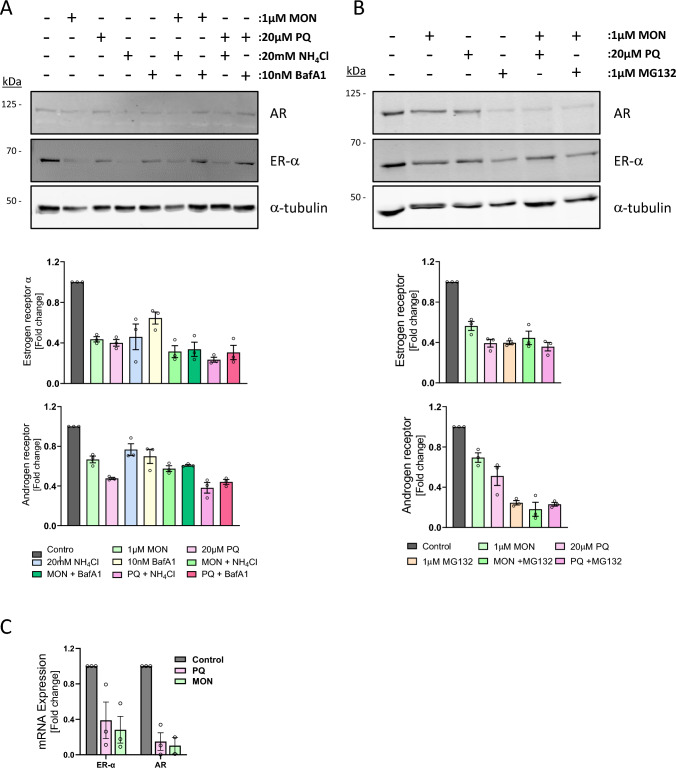
Inhibition of lysosome and proteasomal activity does not rescue endosomal recycling inhibitor-induced downregulation of ER-α and AR. **A** MCF-7 cells were treated with ERIs plus or minus the lysosomal inhibitors NH_4_Cl or BafA1. All drug treatments were for 24 h with the exception of BafA1, which was 4 h. Lysates were immunoblotted with indicated antibodies and the histogram indicates the fold difference in protein levels relative to untreated cells (*n* = 3). **B** MCF-7 cells were treated with endosomal recycling inhibitors plus or minus 1 µM MG132 in serum free medium for 24 h. Lysates were immunoblotted and probed as previously. Bar graph indicates fold difference in protein levels relative to untreated cells (*n* = 3). **C** MCF-7 cells were treated with 20 µM PQ or 1 µM MON for 24 h and anlaysed for *ESR1* and *AR* mRNA expression by quantitative real-time, reverse transcriptase PCR (qRT-PCR). The results (mean ± standard deviation of three biological replicates, each performed in triplicate) are expressed as mRNA expression levels relative to that for control cells

Previous work from our group has shown that inhibition of the endosomal recycling pathway results in the diversion of endocytosed cell surface proteins from the recycling pathway into the degradative pathway, where they are ultimately broken down in lysosomes [11, 21]. To determine if this is also the mechanism by which ER-α and AR are downregulated by PQ and monensin, MCF-7 cells were treated with each endosomal recycling inhibitor alone or in combination with two different lysosomal inhibitors, ammonium chloride and Bafilomycin A1 (BafA1). If the downregulation of ER-α and AR is caused by their degradation in lysosomes, then their protein levels should be restored by addition of lysosomal inhibitors. Unsurprisingly given that ER-α and AR primarily reside in the cytoplasm and not at the plasma membrane, the lysosomal inhibitors had no effect on their downregulation (Fig. 2A). To determine if ER-α and AR are instead degraded by the proteasome, cells were co-treated with the ERIs and the proteasomal inhibitor MG132; however, no restoration of hormone receptor levels was observed (Fig. 2B). Indeed, MG132 alone induced a striking reduction of ER-α and AR protein levels. MG132, as well as other proteasome inhibitors, has been reported to reduce the expression of members of the ERBB family and promote their lysosomal degradation [22].

This family of receptor tyrosine kinases regulates the estrogen-signalling pathway via direct phosphorylation of ER or activation of mitogen-activated protein kinases, which consequently enhances ER signalling [23]. Thus, it can be concluded that the ERI-induced reduction of ER-α and AR protein levels is not due to the lysosomal or proteasomal degradation of these hormone receptors and suggests that the PQ- and monensin-induced downregulation occurs at the transcriptional level. To confirm this, we measured the levels of *ESR1* (gene encoding ER-α) and *AR* mRNA by quantitative reverse-transcription PCR (RT-qPCR). Both PQ and monensin led to a significant reduction in the levels of *ER-α* and *AR* transcripts in MCF-7 cells (Fig. 2C). Thus, the ERI-induced reduction in ER-α and AR occurs at the transcriptional level.

### ER-α downregulation is an indirect consequence of the lysosomal degradation of HER3

**Fig. 3:**
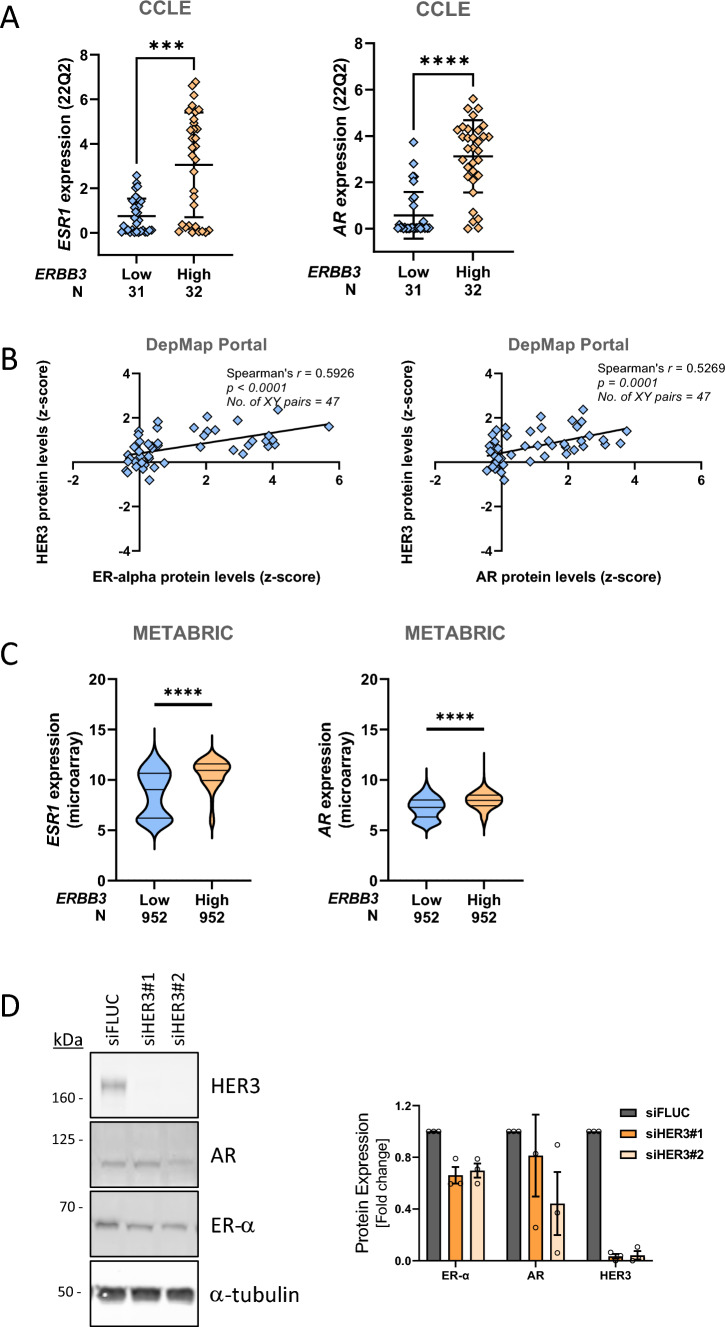
Hormone receptor expression positively correlates with HER3 expression in breast cancer. **A** *ESR1* (left) and *AR* (right) expression positively correlates with *ERBB3* expression in breast cancer cell lines from the Cancer Cell Line Encyclopedia (*N* = 63). Data is equal to mean ± std dev. A Mann–Whitney U statistical analysis test was performed (*** *p* < 0.001). **B** Proteomic analysis of ER-α (left) and AR (right) and HER3 protein levels in breast cancer cell lines available in the DepMap Portal database (*N* = 47). **C** ESR1 (left) and AR (right) expression positively correlates with ERBB3 expression in breast tumours analysed by the METABRIC study and accessible in cBioPortal (*N* = 1903). The violin plot depicts the median and 25th and 75th percentiles, and the whiskers extend to the maximum and minimum values. Data is equal to mean ± std dev. A Mann–Whitney U statistical analysis test was performed (**** *p* < 0.0001). **D** Lysates of MCF-7 cells depleted of HER3 were immunoblotted with the indicated antibodies. The histogram represents the fold difference in protein levels relative to cells transfected with a non-targeting siRNA duplex

It has long been known that the ER-α and AR signalling pathways intersect with the signalling pathways of ERBB family members [24, 25]. HER2 and HER3 have been reported to interact directly with ER-α and mediate its phosphorylation [26], and their signalling is known to influence ER-α and AR gene expression [27–31]. Given that these receptor tyrosine kinases are degraded upon downregulation of the endosomal recycling pathway [11], it is possible that PQ and monensin indirectly downregulate ER-α and AR by promoting the degradation of HER2 and/or HER3. [24, 25]. To investigate this, we analysed publicly available cancer genomics datasets to determine if the expression of HER2 and HER3 is correlated with that of ER-α and AR. First, we stratified the 63 breast cancer cell lines in the Cancer Cell Line Encyclopedia (CCLE) into two groups, one that expresses above median levels of each RTK and the other that expresses below median levels. Both *ESR1* and *AR* mRNA levels were significantly higher in the *ERBB3-*high group (Fig. 3A). Furthermore, there was a positive correlation between ER-α and AR protein levels with HER3 protein levels in the 47 breast cancer cell lines for which protein array data were available (Fig. 3B). We next examined whether this positive correlation could also be observed in the ~ 2,000 breast tumours analysed by the METABRIC study [32]. Again, there was a statistically significant positive correlation between the expression of *ESR1* and *AR* mRNA with *ERBB3* mRNA (Fig. 3C). The correlation between HER2 expression and both hormone receptors was not as consistent. There was a positive correlation between their mRNA expression levels in the breast cancer cell lines and breast tumours, but no significant correlation at the protein level (Fig. S1A–C).

These results indicate in breast cancer that there is a strong and consistent positive correlation between the expression of HER3 and that of ER-α and AR. To confirm this directly, we depleted HER3 in MCF-7 cells and observed an approximately 30% reduction in ER-α protein levels (Fig. 3D). The effect of HER3 depletion on AR was less consistent. These data demonstrate that the expression of HER3 and ER-α and AR is positively correlated and that knockdown of HER3 results in consistent downregulation of ER-α, supporting the hypothesis that the reduction in ER-α and AR levels upon endosomal recycling inhibitor treatment is due to the degradation of HER3 in lysosomes. The effect of HER2 knockdown was not assessed as MCF-7 cells express negligible levels of HER2 protein (Fig. S2A).

To confirm that the ERBB and ER-α signalling pathways interact in MCF-7 cells, serum-starved cells were stimulated with E2 estradiol to activate ER-α, or EGF, and HRGβ to activate EGFR and HER3, respectively. Both E2 and HRGβ induced the phosphorylation of ER-α, as determined by a mobility shift of the protein observed by Western blot. HRGβ, but not E2, induced the phosphorylation of HER3 (Fig. S2B). These results indicate that in MCF-7 cells HER3 can activate ER-α, but ER-α does not activate HER3, and supports the hypothesis that HER3 regulates the expression and activation of ER-α, but not vice versa.

### Endosomal recycling inhibitors synergise with tamoxifen

Given that PQ and monensin indirectly downregulate two hormone receptors that are commonly amplified in BC, we next investigated if they could be combined with commonly used HR-targeting therapies. Tamoxifen is a selective ER modulator widely used to treat ER-positive breast cancer [33]. To determine if the ERIs synergise with tamoxifen, MTT cell viability assays were performed. Synergy occurs when a combination of two or more drugs has a greater effect than that expected from the sum of the effects of the drugs when used individually [34]. MCF-7 cells were treated with increasing concentrations of the ERI alone, tamoxifen alone, or the combination of both drugs, for 72 h. The results indicated that the combination had a greater inhibitory effect on cell viability than either drug alone (Table 1 and Fig. S3). Using the Chou-Talalay method [15], a combination index (CI) of 0.83 was calculated for PQ and tamoxifen in MCF-7 cells, indicating that these two drugs synergise. CI values less than 1 indicate synergy, and CI values greater than 1 indicate antagonism. A stronger synergy was observed between monensin and tamoxifen. Strong synergy between tamoxifen and PQ was also observed in the invasive lobular carcinoma MDA-MB-134 VI cell line which has de novo resistance to tamoxifen [35]. Surprisingly, PQ and tamoxifen displayed antagonism in SUM44-PE cells (Table 1).

**Table 1:** IC50 values for the ER-α antagonist tamoxifen, endosomal recycling inhibitors (primaquine and monensin) and their combinations in the indicated breast cancer cell lines

Cell line	Tamoxifen (µmol/L)	Primaquine (µmol/L)	Monensin (µmol/L)	Effect (% Death)	Synergy (CI)
**MCF7**	5.2 ± 0.3			50	
		17.4 ± 1.5		50	
*Combination*	2.2	10.2		65	0.83
**MCF7**	5.4 ± 1.4			50	
			0.44 ± 0.08	50	
*Combination*	1.25		0.25	60	0.53
**MDA-MB-143 VI**	5.9 ± 0.8			50	
		38.9 ± 2.2		50	
*Combination*	2.4	23.9		55	0.62
**SUM44-PE**	8.4 ± 2.2			50	
		82.1 ± 17.9			
*Combination*	4.3	43.0		50	1.4

Combination indexes (CI) were determined by the Chou and Talalay method. CI < 1 = synergy; CI = 1 additivity; CI > 1 = antagonism. The combination row indicates the concentration of each drug used in combination to achieve the indicated percentage cell death and CI value. See Fig. S3 for representative drug response curves

**Fig. 4:**
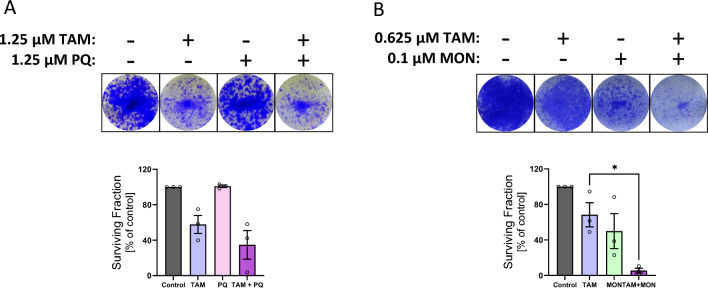
Endosomal recycling inhibitors synergise with hormone receptor antagonists. **A**, **B** Clonogenic assays of MCF-7 cells individually or dual treated with the indicated concentrations of tamoxifen and either PQ or MON for 10 days. Histogram indicates the surviving cells expressed as a percentage of the untreated control (*n* = 3)

These findings were confirmed using clonogenic assays. MCF-7 cells were treated with low doses of the drugs for 10 days, and surviving cells were stained with crystal violet and quantified. In contrast to the cell viability assay, the combination of tamoxifen and PQ did not perform better than tamoxifen alone (Fig. 4A). However, combining monensin with tamoxifen resulted in much greater cytotoxicity than either drug alone (Fig. 4B).

Taken together, these results demonstrate that endosomal recycling inhibitors can modulate the levels of the nuclear receptors ER-α and AR, and that they may be effective at treating certain forms of breast cancer when used in combination with hormone receptor antagonists.

## Discussion

Tamoxifen, a drug which inhibits the estrogen receptor, has been used for decades to treat ER-positive breast cancer. Although initially effective, long-term use of tamoxifen has led to approximately 40% of patients developing acquired resistance to this therapy [36]. Thus, new drugs and drug combinations are urgently required that target ER and AR, to overcome this drug resistance.

A novel approach to treating cancer could involve inhibition of the endosomal recycling pathway. Defective endosomal recycling has been implicated in the development and progression of many cancer types, including breast and prostate cancer, and since this pathway is frequently hyperactivated in cancer, a therapeutic dose of an endosomal recycling inhibitor is less likely to affect non-malignant cells [12]. We and others have shown that clinically relevant cell surface proteins such as EGFR, HER2, HER3, N-cadherin, and c-Met, are downregulated when endosomal recycling is inhibited [11, 21, 37–40].

There are several small molecules that inhibit the endosomal recycling pathway, including PQ and monensin [20, 41], and we recently reported that PQ blocks the recycling of HER2 and HER3 back to the plasma membrane in HER2-positive breast cancer [11]. To gain a greater understanding of the mechanism of action of PQ, we performed a reverse-phase protein array assay using BT474 HER2-positive BC cells to identify proteins and signalling pathways that are altered by PQ treatment. Among the top 30 hits were the hormone receptors ER-α and AR, both of which were downregulated by PQ. We validated these findings by Western blot and then moved to investigate if PQ and monensin could also downregulate ER-α and AR in other BC cancer cell lines. We observed a dose-dependent reduction of the hormone receptors in all BC cell lines tested. Further, we have recently reported similar effects in prostate cancer cells [42].

PQ downregulates HER3 by blocking its trafficking back to the plasma membrane and diverting it to lysosomes where it is degraded. The PQ-induced downregulation of HER3 can be rescued by cotreating cells with a lysosomal inhibitor [11]. However, we were unable to restore hormone receptor levels with lysosomal or proteasomal inhibitors, suggesting that the effect of the ERIs on these proteins occurs by a different mechanism. Quantitative reverse transcriptase PCR showed that the downregulation occurred at the transcriptional level, likely as a downstream consequence of the lysosomal degradation of HER3.

ERBB and hormone receptor signalling pathways overlap, and approximately two thirds of HER2 + breast cancers also express hormone receptors. The presence of ER-α influences the response to HER2-targeted therapies while HER2 expression impacts the efficacy of endocrine therapies [13]. Given this crosstalk, we reasoned that there may be a feedback loop that regulates the expression of components of these pathways. MCF-7 cells express high levels of HER3 and minimal levels of EGFR and HER2 (Fig. S2A and [11]), and we observed that both PQ and monensin downregulate HER3 protein levels in these cells (not shown). To investigate if the downregulation of ER-α and AR is an indirect consequence of the ERIs inducing the lysosomal degradation of HER3, we explored cancer genomics databases to determine if there is a correlation between HER3 and hormone receptor expression. We observed a strong positive correlation between HER3 and both ER-α and AR expression in breast cancer cell lines and breast tumours. Furthermore, knockdown of HER3 resulted in a consistent and reproducible reduction in ER-α levels. The effect on AR was more variable. These findings suggest that HER3 regulates ER-α and possibly AR gene expression, and that the effect of PQ and monensin on the hormone receptors is a downstream consequence of the lysosomal degradation of HER3.

HER3 expression has been implicated in resistance to tamoxifen. A study of more than 400 patients with tamoxifen-treated ER-positive breast cancer found that patients whose tumours were also positive for HER2 and HER3 are more likely to relapse while on tamoxifen than those with HER2- and HER3-negative tumours [43]. In addition, siRNA-mediated knockdown of HER3 sensitises breast cancer cell lines to tamoxifen [44]. We investigated whether indirectly downregulating HER3 by inhibiting its endosomal recycling would enhance the efficacy of tamoxifen. Using cell viability and clonogenic assays, we observed that ERIs synergised with tamoxifen in a number of BC cells, including the tamoxifen-resistant invasive lobular carcinoma MDA-MB-134 VI cell line.

Primaquine was approved by the FDA in 1952 to treat patients with malaria and is, thus, a readily available and cost-effective drug with a good safety profile [45]. Therefore, we believe that PQ has the potential to be repurposed as a combination treatment to enhance the efficacy of hormone receptor antagonists and to reduce the emergence of drug resistance. Monensin is a sodium ionophore approved for use as a veterinary medication with a narrow therapeutic window. The FDA has not approved it for use in humans as monensin intoxication can lead to renal failure, rhabdomyolysis, and cardiac failure [46]. Nevertheless, many studies in recent years have demonstrated its potential as a cancer therapeutic [47–51]. Indeed, monensin has been previously reported to reduce the expression of AR in prostate cancer cell lines and to synergise with the anti-androgen flutamide [52]. We have found that ERIs also downregulate AR and synergise with enzalutamide in prostate cancer [42]. Given that monensin displayed strong effects at nanomolar concentrations, with further research and possible modification of its chemical structure monensin could also have clinical utility for the treatment of hormone receptor-positive cancer patients. Since the ERIs do not act directly on the hormone receptors, tumour cells are less likely to develop resistance to these drugs.

### Supplementary Information

Below is the link to the electronic supplementary material.

Supplementary Figure 1. Correlation of hormone receptor and HER2 expression in breast cancer. A ESR1 (left) and AR (right) expression positively correlates with ERBB2 expression in breast cancer cell lines from the Cancer Cell Line Encyclopedia (N = 68). Data equals mean ± std dev. A Mann-Whitney U statistical analysis test was performed (*p < 0.05, **p < 0.01). B Proteomic analysis of ER-α (left), AR (right) and HER2 protein levels in breast cancer cell lines available in the DepMap Portal database (N = 47). C ESR1 (left) and AR (right) expression positively correlates with ERBB2 expression in breast tumours analysed by the METABRIC study and accessible in cBioPortal (N = 1866). The violin plot depicts the median and 25th and 75th percentiles and the whiskers extend to the maximum and minimum values. Data equals mean ± std dev. A Mann-Whitney U statistical analysis test was performed (**** p < 0.0001). Supplementary Figure 2. HER3 and ER-α signalling pathways intersect. A Western blot analysis of HER2, HER3, AR and ER-α protein expression levels in lysates of the indicated breast cancer cell lines. B MCF-7 cells were serum-starved for 24 hours and then stimulated with the indicated growth factor or hormone for 30 minutes. Lysates were immunoblotted with the indicated antibodies. Supplementary Figure 3. Representative dose response curves for the tamoxifen plus ERI combinations in the indicated cell lines. (PDF 369 kb)

## Data Availability

All datasets are available upon request (a.lindsay@ucc.ie).
