# Supplementary figures and images for: Endosomal recycling inhibitors downregulate estrogen receptor-alpha and synergise with endocrine therapies
Source: Breast Cancer Res Treat. 2024 Jan 16;204(3):631–42. doi: 10.1007/s10549-023-07225-2 (PMC10959794; doi:10.1007/s10549-023-07225-2)

Figure S1

A

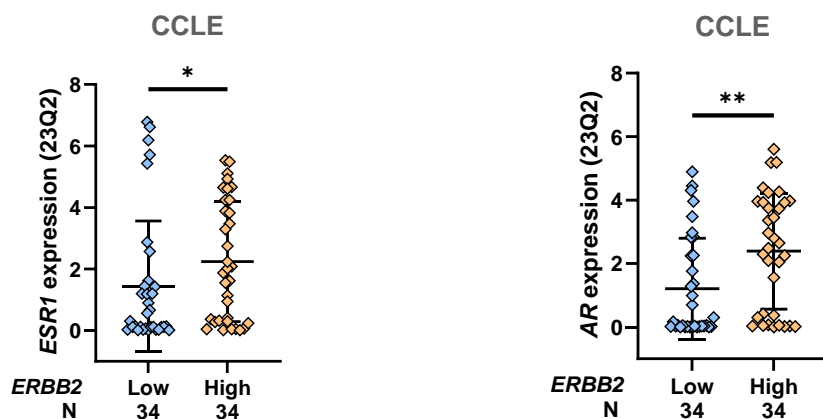

B

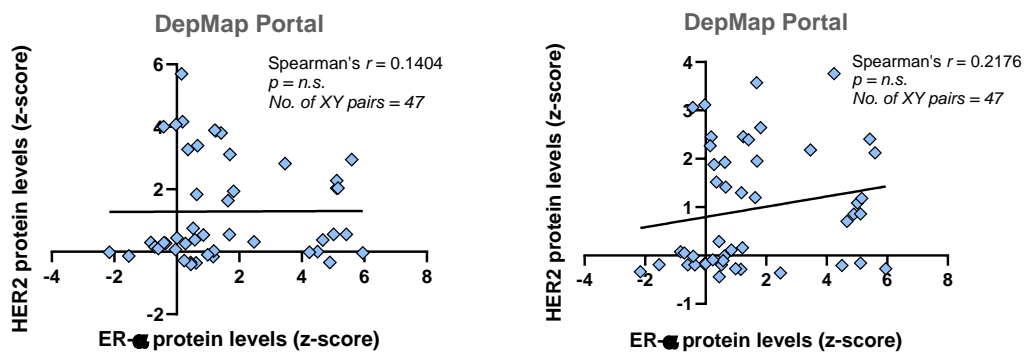

C

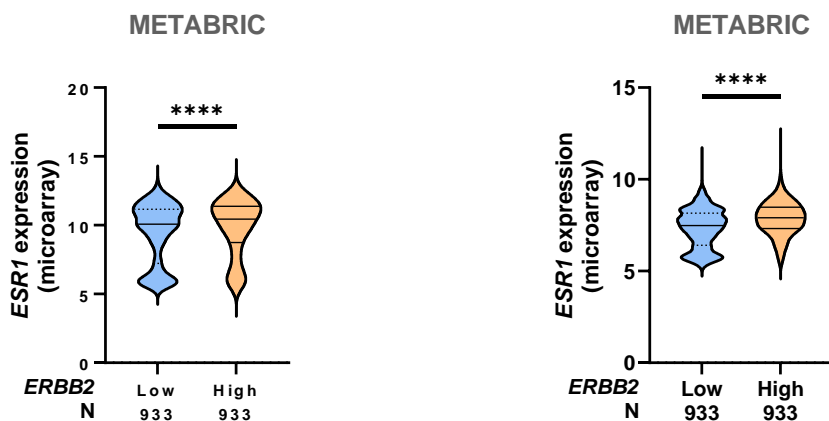

Figure S2

A

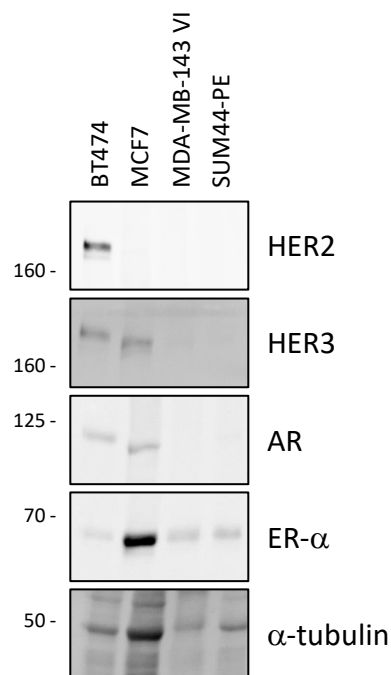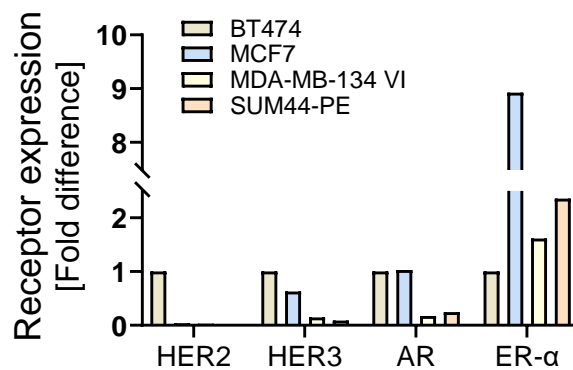

B

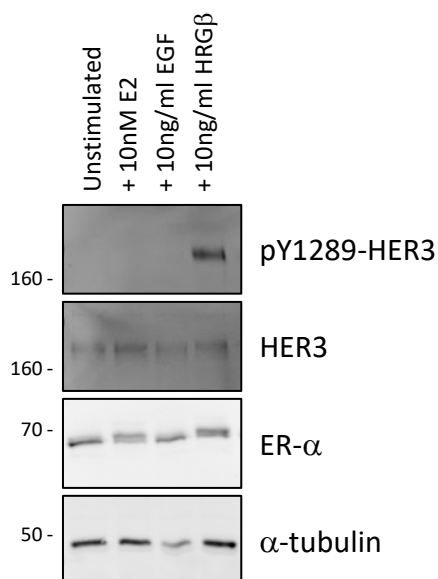

Figure S3

MCF-7

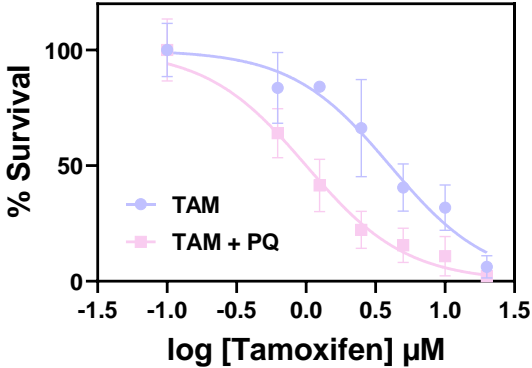

MCF-7

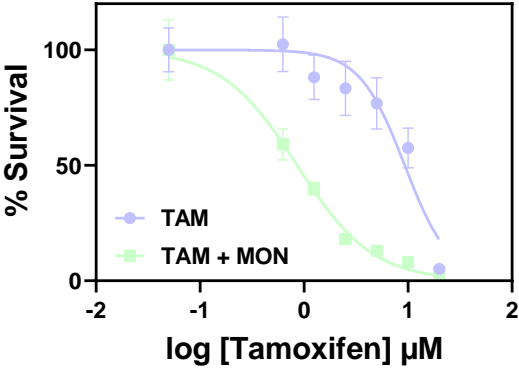

MDA-MB-134 VI

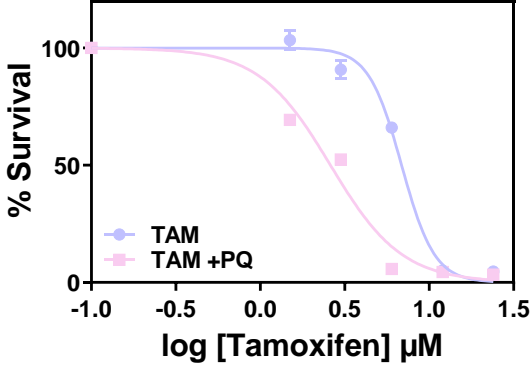

SUM44-PE

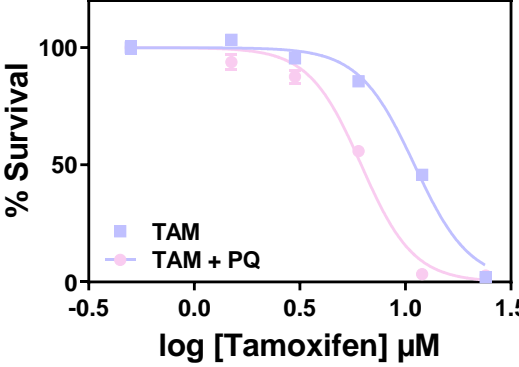

Supplement: Supplementary file 1 — Supplementary Figure 1. Correlation of hormone receptor and HER2 expression in breast cancer. A ESR1 (left) and AR (right) expression positively correlates with ERBB2 expression in breast cancer cell lines from the Cancer Cell Line Encyclopedia (N = 68). Data equals mean ± std dev. A Mann-Whitney U statistical analysis test was performed (*p < 0.05, **p < 0.01). B Proteomic analysis of ER-α (left), AR (right) and HER2 protein levels in breast cancer cell lines available in the DepMap Portal database (N = 47). C ESR1 (left) and AR (right) expression positively correlates with ERBB2 expression in breast tumours analysed by the METABRIC study and accessible in cBioPortal (N = 1866). The violin plot depicts the median and 25th and 75th percentiles and the whiskers extend to the maximum and minimum values. Data equals mean ± std dev. A Mann-Whitney U statistical analysis test was performed (**** p < 0.0001). Supplementary Figure 2. HER3 and ER-α signalling pathways intersect. A Western blot analysis of HER2, HER3, AR and ER-α protein expression levels in lysates of the indicated breast cancer cell lines. B MCF-7 cells were serum-starved for 24 hours and then stimulated with the indicated growth factor or hormone for 30 minutes. Lysates were immunoblotted with the indicated antibodies. Supplementary Figure 3. Representative dose response curves for the tamoxifen plus ERI combinations in the indicated cell lines. (PDF 369 kb) [file 10549_2023_7225_MOESM1_ESM.pdf]
